# What Is the Evidence That the Tissue Doppler Index E/e′ Reflects Left Ventricular Filling Pressure Changes After Exercise or Pharmacological Intervention for Evaluating Diastolic Function? A Systematic Review

**DOI:** 10.1161/JAHA.116.004766

**Published:** 2017-03-15

**Authors:** Oleg F. Sharifov, Himanshu Gupta

**Affiliations:** ^1^ Department of Medicine University of Alabama at Birmingham AL; ^2^ VA Medical Center Birmingham AL; ^3^ Cardiovascular Associates of the Southeast Birmingham AL

**Keywords:** diastolic dysfunction echocardiography, diastolic heart failure, Doppler echocardiography, E/e′, exercise echocardiography, left ventricular diastolic dysfunction, left ventricular diastolic function, left ventricular filling pressure, Echocardiography, Heart Failure, Diagnostic Testing, Exercise Testing, Clinical Studies

## Abstract

**Background:**

Noninvasive echocardiographic tissue Doppler assessment (E/e′) in response to exercise or pharmacological intervention has been proposed as a useful parameter to assess left ventricular (LV) filling pressure (LVFP) and LV diastolic dysfunction. However, the evidence for it is not well summarized.

**Methods and Results:**

Clinical studies that evaluated invasive LVFP changes in response to exercise/other interventions and echocardiographic E/e′ were identified from PubMed, Scopus, Embase, and Cochrane Library databases. We grouped and evaluated studies that included patients with preserved LV ejection fraction (LVEF), patients with mixed/reduced LVEF, and patients with specific cardiac conditions. Overall, we found 28 studies with 9 studies for preserved LVEF, which was our primary interest. Studies had differing methodologies with limited data sets, which precluded quantitative meta‐analysis. We therefore descriptively summarized our findings. Only 2 small studies (N=12 and 10) directly or indirectly support use of E/e′ for assessing LVFP changes in preserved LVEF. In 7 other studies (cumulative N=429) of preserved LVEF, E/e′ was not useful for assessing LVFP changes. For mixed/reduced LVEF groups or specific cardiac conditions, results similar to preserved LVEF were found.

**Conclusions:**

We find that there is insufficient evidence that E/e′ can reliably assess LVFP changes in response to exercise or other interventions. We suggest that well‐designed prospective studies should be conducted for further evaluation.

## Introduction

Left ventricular diastolic dysfunction leading to heart failure with preserved ejection fraction (HFpEF) is a major clinical problem.^1^, ^2^, ^3^ Elevated left ventricular filling pressure (LVFP) is often used as a clinical surrogate for impaired diastolic function in patients with preserved left ventricular ejection fraction (LVEF).^4^, ^5^ LVFP is usually measured at rest in routine clinical practice. However, changes in LVFP with exercise or other physiological intervention provide incremental information to assess diastolic function.^5^, ^6^, ^7^, ^8^, ^9^, ^10^ A direct measurement of LVFP requires an invasive intervention, which has significant risk and costs, and is therefore performed in select patients only. Echocardiography is frequently used for noninvasive evaluation of diastolic function and estimating LVFP.^4^, ^5^, ^6^ Echocardiographic quantification of LVFP is based on E/e′ measurement, which is the ratio of the early diastolic velocity on transmitral Doppler (E) and the early diastolic velocity of mitral valve annulus obtained from tissue Doppler (e′).^4^, ^5^, ^6^, ^11^, ^12^, ^13^, ^14^ The guidelines recommend using E/e′ in evaluating LV diastolic function.^4^, ^5^, ^6^, ^10^ In research studies, E/e′ is also used as a primary or secondary end point for assessing the treatment efficacy and quantifying changes in LVFP.^11^, ^12^, ^13^, ^14^, ^15^, ^16^, ^17^, ^18^, ^19^

Despite extensive use of E/e′, there continues to be ongoing debate about the usefulness of E/e′ in assessing LVFP.^20^, ^21^, ^22^, ^23^, ^24^ In our recent comprehensive meta‐analysis, we have found limited evidence for the use of E/e′ under resting conditions to estimate LVFP in preserved LVEF.^20^ It has been suggested that changes in E/e′ with exercise or other physiological/pharmacologic interventions may more accurately reflect changes in the LVFP and diastolic properties.^5^, ^6^, ^8^, ^10^, ^25^ Here we decided to evaluate the evidence describing the relationship of E/e′ and LVFP in preserved LVEF with exercise or other physiological interventions. We also summarize the available evidence describing the relationship of E/e′ and LVFP in a wider spectrum of LVEF and for specific cardiac conditions.

## Methods

### Search Strategy and Study Selection

Original clinical studies that evaluated LVFP by using echocardiographic E/e′ and invasive techniques were screened from PubMed, Scopus, Embase, and Cochrane Library databases to September 2016 using a number of search strategies (Figure). Specific search terms and full‐text studies excluded after evaluation are listed in Tables S1 and S2. Clinical studies (in English) that reported changes in E/e′ and invasively measured LVFP attributable to physiologic and/or pharmacologic or other therapeutic intervention and/or repeated serial measurements in the adult subjects (age >18 years) with any LVEF and clinical conditions were included. References of important studies were also reviewed for comprehensive search. LVFP measurements included LV end diastolic pressure, LV pre‐A wave pressure, LV mean diastolic pressure, left atrial pressure, and pulmonary capillary wedge pressure (PCWP) obtained during the left or right heart catheterization or from a permanently implantable cardiac pressure monitoring system. Only studies that utilized transthoracic echocardiographic pulsed‐wave tissue Doppler imaging for E/e′ measurements at interventricular septum (E/e′_septal_), lateral mitral annulus (E/e′_lateral_), and/or mean of septal and lateral values (E/e′_mean_) were selected.

**Figure 1:**
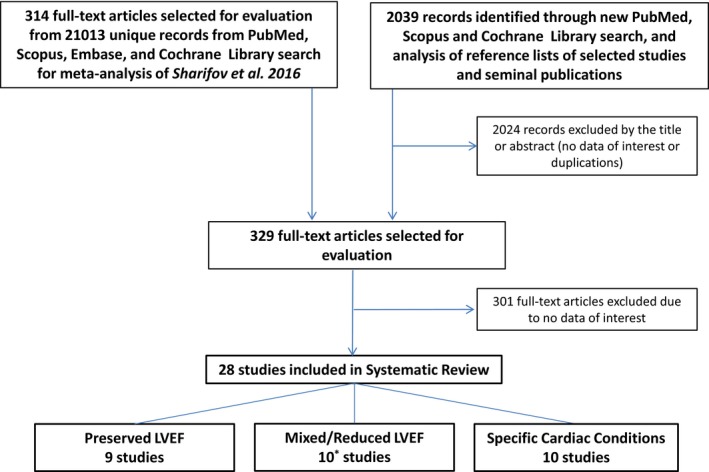
Summary of the literature search. Studies that include data for patients with preserved LVEF (LVEF ≥50%) were our primary interest. Other studies that include data for patients with mixed or reduced LVEF (LVEF <50%) and patients with specific cardiac conditions were our secondary interest. For this review, with studies identified during a comprehensive literature search for recent meta‐analysis, Sharifov et al^20^ were initially evaluated. An updated literature search was then performed based on specific search strings as described. One study included a data set for primary and secondary analysis. LVEF indicates left ventricular ejection fraction.

The studies were included if they reported at least 1 of the following data sets: (1) E/e′ and LVFP values at baseline and after intervention; (2) changes in E/e′ and LVFP values because of intervention; (3) assessment of correlation between E/e′ and LVFP postintervention, alone, or combined with baseline; (4) assessment of correlation between changes in E/e′ and LVFP with intervention; and (5) the diagnostic accuracy of either postintervention E/e′ values or postintervention changes in E/e′ to predict elevated LVFP or LVFP changes.

### Patient Cohorts and Study Analysis

Included studies were grouped and analyzed based on patient cohorts. The first group was for studies that included patients with LVEF ≥50%, including HFpEF patients, but without a substantial number of moderate‐to‐severe valvular heart disease, hypertrophic or restrictive cardiomyopathy, congenital heart disease, acute coronary syndromes, septic shock, cardiac transplant, and atrial fibrillation. This group was our primary interest. Other groups were for studies that included patients with reduced/mixed LVEF, and for studies that included patients grouped with specific cardiac conditions (eg, cardiac transplants). Overall, we found 28 studies: 9 studies^24^, ^26^, ^27^, ^28^, ^29^, ^30^, ^31^, ^32^, ^33^ for our primary interest, and 19 studies^25^, ^34^, ^35^, ^36^, ^37^, ^38^, ^39^, ^40^, ^41^, ^42^, ^43^, ^44^, ^45^, ^46^, ^47^, ^48^, ^49^, ^50^, ^51^ for secondary interest (Figure). One study included a data set for primary and secondary interest.^30^ Since most of the studies were single center with differing methodologies with many reporting only a limited data set, we chose descriptive methodologies to summarize the results.

## Results

### Studies in LVEF ≥50% With or Without HFpEF

Table 1 summarizes study details and results for the 9 studies that included participants with preserved LVEF (≥50%), including HFpEF patients (see Table S3 for more details). All studies, except 1,^29^ had a prospective design and all studies, except 1,^32^ simultaneously measured echocardiographic and hemodynamic variables. Most of these studies had a low sample size (median N = 22 with interquartile range of 11–82). Three of these studies had subjects perform exercise stress echocardiography using a supine bicycle^29^, ^31^ or passive and then active leg‐raise^33^ for evaluating patients with suspected HFpEF. There was an increase in invasive LVFP but no consistent relationship for the changes in E/e′ postintervention in these 3 studies. Talreja et al^31^ found that E/e′ provides a reliable estimation of PCWP with exercise in a small study of 12 patients. Based on their scatterplot,^31^ we estimated that stress E/e′_septal_ >15 predicts PCWP ≥20 mm Hg with sensitivity of 83% and specificity of 100%. Maeder et al^29^ found decreased E/e′_septal_ with exercise and no correlation between poststress E/e′_septal_ and PCWP. In the largest exercise study in patients with exertional dyspnea (N=181), Choi et al^33^ recorded no change of E/e′_septal_ despite a significant elevation of LV end diastolic pressure with passive and active leg raise.

**Table 1:** Summary of Studies With Subjects With Preserved LVEF (>50%), With or Without HFpEF Patients

Study	Study Design	Population	N	Intervention	Echo./Cath. Timing	LVFP Change Postintervention	E/e′ Change Postintervention	E/e′‐LVFP Relation Postintervention	ΔE/e′‐ΔLVFP Relation	Prediction of Elevated LVFP Postintervention	Study Summary for Relationship Between E/e′ and LVFP	Comments
Interventions to increase LVFP												
Firstenberg, 2000^28^	Prospective^a^	Healthy volunteers	7	Saline infusion	Simultaneous	↑ PCWP	↔ Lateral, Septal	···	···	···	···	E/e′ does not change despite LVFP increase
Talreja, 2007^31^	Prospective	Exertional dyspnea (NYHA class II–III)	12	Supine bicycle	Simultaneous	↑ PCWP	↑ Septal	···	···	Se./Sp.^b^: 83%/100% to predict PCWP ≥20 mm Hg if E/e′>15	E/e′ does provide a reliable estimation of PCWP with exercise (E/e′ >15 is associated with PCWP >20 mm Hg)	
Maeder, 2010^29^	Case–Control^a^	14 HFpEF and 8 matching Controls	22	Supine bicycle	Simultaneous	↑ PCWP	↓ Septal	n.s.	···	···	E/e′ does not reflect the hemodynamic changes during exercise in HFpEF patients and in controls	
Choi, 2016^33^	Prospective	HFpEF (at rest 8<E/e′<15, E/A<1, or e′<8 cm/s)	181	Passive and active leg‐raise	Simultaneous	↑ LVEDP, Pre‐A	↔ Septal	···	···	···	···	E/e′ does not change despite LVFP increase
Interventions to decrease LVFP												
Firstenberg, 2000^28^	Prospective^a^	Healthy volunteers	7	Lower‐body negative pressure	Simultaneous	↓ PCWP	↔ Lateral, Septal	···	···	···	···	E/e′ does not change despite LVFP decrease
Efstratiadis, 2009^32^	Prospective	HFpEF patients	10	Nesiritide i.v.	Consequentive	↓ LVEDP, PCWP	↓ Lateral	···	···	···	···	See also Weeks, 2008^45^
Chan, 2011^27^	Prospective	Patients without significant CAD	16	Dobutamine i.v.	Simultaneous	↓ LVEDP, LVMDP	↔ Lateral, Septal	n.s.	···	···	E/e′ does not predict changes in LVFP at peak stress with dobutamine	
Manouras, 2013^30^	Prospective^a^ Consecutive	Stable angina and/or exertional dyspnea	38	Nitroglycerin i.v.	Simultaneous	↓ LVEDP, Pre‐A	↔ Lateral, Septal, Mean	n.s.	n.s.	···	E/e′ does not reliably predict changes in LVFP; not recommended for monitoring load reducing therapy	Results for cohort with LVEF >55%
Santos, 2015^24^	Prospective	Unexplained dyspnea	118	Position change from supine to upright	Simultaneous	↓ PCWP	↔ Lateral, Septal, Mean	n.s.	n.s.	···	E/e′ does not accurately estimate PCWP. Positional change in E/e′ does not reflect change in PCWP	
Analysis of combined measurements from baseline and during intervention												
Firstenberg, 2000^28^	Prospective^a^	Healthy volunteers	7	Lower‐body negative pressure—saline infusion	Simultaneous	↓↔↑ PCWP	↔ Lateral, Septal	n.s.	···	···	···	E/e′ does not change despite wide range of LVFP change
Bhella, 2011^26^	Prospective	11 outpatient HFpEF, 24 old and 12 young healthy Controls	47	Lower‐body negative pressure—saline infusion	Simultaneous	↓↔↑ PCWP	··· Mean	···	···	···	E/e′ does not reliably track changes in LVFP; not recommended in research with healthy volunteers or for the titration of therapy in HFpEF patients	*R* ^2^ and Slopes for individual linear regression widely differed

↑ or ↓ indicates statistically significant increase or decrease was measured in the cohort; ↔, no statistically significant change was measured in the cohort; CAD, coronary artery disease; HFpEF, heart failure with preserved ejection fraction; Lateral, Septal, and Mean, E/e′_lateral_, E/e′_septal_, and E/e′_mean_; LVEDP, left ventricular end diastolic pressure; LVEF, left ventricular ejection fraction; LVFP, left ventricular filling pressure; LVMDP, left ventricular mean diastolic pressure; N, number of patients with LVEF >50% (not always a total N of patients in the study); n.s., study reports that correlation coefficient is not statistically significant; NYHA, New York Heart Association; PCWP, pulmonary capillary wedge pressure; pre‐A, left ventricular pre‐A wave pressure; Se./Sp., Sensitivity and Specificity.

a Based on our read.

b Our assessment made from the study data.

In another set of studies, authors performed stress echocardiography using differing pharmacological interventions^27^, ^30^, ^32^ or body position change^24^ that resulted in significant decrease of LVFP (Table 1). Only in 1 small study,^32^ authors reported the decrease of group average E/e′_lateral_ in response to decreased LVFP for 10 HFpEF patients. However, this study did not provide any individual data for further analysis. Interestingly, in another publication from the same group^45^ (Table 2), authors reported no correlation between individual changes of E/e′ and LVFP for a combined cohort of 10 HFpEF and 15 heart failure with reduced ejection fraction (HFrEF) patients. In studies^24^, ^27^, ^30^ with a total of 179 HFpEF and/or coronary artery disease patients, there were no significant changes in E/e′ values despite reduced LVFP. Furthermore, in these studies there was no significant correlation between postintervention values of E/e′ and LVFP or between individual changes in E/e′ and LVFP.

**Table 2:** Summary of Studies With Subjects With Reduced or Various LVEF, With or Without HF

Study	Study Design	Population	N	Intervention	Echo./Cath. Timing	LVFP change Postintervention	E/e′ Change Postintervention	E/e′‐LVFP Relation Postintervention	ΔE/e′‐ΔLVFP Relation	Prediction of Elevated LVFP Postintervention	Study Summary for Relationship Between E/e′ and LVFP	Comments
Interventions to increase LVFP												
Burgess, 2006^25^ Gibby, 2013^44^	Prospective^a^	Unselected patients undergoing heart catheterization, LVEF 56±12%	37	Single‐leg supine cycle	Simultaneous	↔(?)^b^ LVMDP	↔(?)^b^ Septal	Sign.	···	To detect LVMDP >15 mm Hg: *AUC:* 0.89^c^; Se./Sp.: 73%/96% if E/e′ >13 in^25^ Se/Sp*.:* 67%/95% if E/e′ >13 in^44^	E/e′ does correlate with LVFP during exercise and it can be used to reliably identify patients with elevated LVFP during exercise	LVMDP and E/e′ significantly increased in 9 patients during exercise
Yamada, 2014^46^	Consecutive	Various chronic cardiac diseases, LVEF 58±14%	22	Leg‐positive pressure	Simultaneous	↑ LVEDP. Pre‐A	↑ Lateral	···	···	···	···	On group average, E/e′ does increase reflecting elevation of LVFP
Marchandise, 2014^40^	Prospective Consecutive	LV systolic dysfunction, LVEF 27±11%	40	Semisupine bicycle	Simultaneous	↑ PCWP	↓ Lateral, Septal, Mean	Sign.	···	···	E/e′ is less reliable for estimating LVFP during exercise than at rest	
Interventions to decrease LVFP												
Weeks, 2008^45^	Prospective	10 HFpEF and 15 HFrEF, LVEF 45±10%	25	Nesiritide i.v.	Consequentive	↓ LVEDP, PCWP	↓ Lateral	···	n.s.	···	E/e′ does not reflect changes in LVFP	See also Efstratiadis, 2009^32^
Manouras, 2013^30^	Prospective^a^ Consecutive	Stable angina and/or exertional dyspnea, LVEF >40%	65	Nitroglycerin i.v.	Simultaneous	↓ LVEDP, Pre‐A	↔ Lateral, Septal, Mean	n.s.	n.s.	To detect LVEDP >16 mm Hg: AUC n.s. To detect Pre‐A >12 mm Hg: AUC n.s.	E/e′ does not reliably predict changes in LVFP	
Egstrup, 2013^36^	Prospective^a^	Chronic HFrEF, LVEF 36±8%	14	Dobutamine i.v.	Simultaneous	↔ PCWP	↔ Septal	n.s.	···	···	E/e′ does not reflect the PCWP during low‐dose dobutamine	
Chiang, 2014^42^	Prospective^a^ Consecutive	Suspected CAD, LVEF 43±16%	60	Glyceryl trinitrate i.v.	Simultaneous	↓ LVEDP, Pre‐A	↓ Septal	n.s.	···	···	E/e′ does not reflect changes in LVFP	
Serial or repeated measurements												
Ritzema, 2011^41^	Sub analysis of prospectively enrolled clinical trial cohort	Ambulant chronic HFrEF, LVEF 32±12%	15	1 to 7 measurements (median 4) for 0 to 52 weeks (median 23 weeks) using implantable LAP monitoring system	Simultaneous	↓↑ LAP	↓↑ Lateral, Septal, Mean	For total of 60 measurements Lateral: n.s. Septal: Sign. Mean: n.s.	Septal: Sign.	For total of 60 measurement: to detect LAP ≥15 mm Hg: Lateral AUC 0.90^c^; Se./Sp*.:* 73%/87% if E/e′≥12 Septal AUC 0.90^c^; Se./Sp.: 84%/91% if E/e′≥15 Mean AUC 0.95^c^; Se./Sp.: 84%/96% if E/e′≥14	While E/e′ weakly correlate with LAP, E/e′ does reliably detect raised LAP	
Goebel, 2011^50^	Sub analysis of prospectively enrolled clinical trial cohort	Patients scheduled for aortocoronary bypass surgery, LVEF between 25% and 35%	5	Repeated measurements for 6 months using a telemetric intraventricular pressure sensor	Simultaneous	↓↑ LVEDP, LVMDP	↓↑ Lateral, Septal, Mean	For total of 21 measurements Lateral: n.s. Septal: n.s. Mean: n.s.	···	For total of 21 measurements: to detect LVEDP >15 mm Hg: AUC n.s. to detect LVMDP >12 mm Hg: Lateral, Septal AUC n.s.; Mean AUC 0.82^c^	E/e′ does not reliably correlate with LVFP, does not reliably detect raised LVFP	

? indicates not clear from text; ↑ or ↓, statistically significant increase or decrease was measured in the cohort; ↔, no statistically significant change was measured in the cohort; AUC, area under the receiver operating characteristic curve; CAD, coronary artery disease; HFpEF/HFrEF, heart failure with preserved/reduced ejection fraction; LAP, left atrial pressure; Lateral, Septal, and Mean, E/e′_lateral_, E/e′_septal_, and E/e′_mean_; LVEDP, left ventricular end diastolic pressure; LVEF, left ventricular ejection fraction; LVFP, left ventricular filling pressure; LVMDP, left ventricular mean diastolic pressure; N, number of patients; PCWP, pulmonary capillary wedge pressure; pre‐A, left ventricular pre‐A wave pressure; Se./Sp., sensitivity and specificity; Sign./n.s., study reports that correlation coefficient is/is not statistically significant.

a Based on our read.

b Our assessment made from the study data.

c Statistically significant value of AUC.

In 2 other studies, participants underwent preload changes leading to lower LVFP caused by low body negative pressure and increase in LVFP by saline infusion.^26^, ^28^ Both studies found that E/e′ cannot reliably track changes in LVFP in healthy people^26^, ^28^ and in HFpEF patients.^26^

### Studies in Reduced or Mixed LVEF

Table 2 summarizes study details and results for the 10 studies that included participants with mixed or reduced LVEF (see Table S4 for more details). In the study of Burgess et al,^25^ which included 37 unselected patients with varying LVEF, authors reported a significant correlation (*r*=0.59) between E/e′_septal_ and LV mean diastolic pressure during single‐leg supine exercise. They reported high AUC value (0.89) for exercise E/e′_septal_ to predict an elevation of LV mean diastolic pressure >15 mm Hg.^25^ In their reports for the same patient cohort, E/e′_septal_ >13 had sensitivity of ≈70% and specificity of ≈95% for estimating elevated LV mean diastolic pressure >15 mm Hg.^25^, ^44^ In another study of 22 patients,^46^ mean E/e′_lateral_ increased with preload stress. However, on detailed analysis, E/e′_lateral_ increase was observed in only a small subset of patients (N=6). No correlation of E/e′ and LVFP or diagnostic value of E/e′_lateral_ was reported.^46^ In another study in patients with reduced LVEF (N=40), authors reported a significant correlation between exercise E/e′ and LVFP and a paradoxical decrease of exercise E/e′ values despite LVFP elevation.^40^

In 4 studies, investigators used different pharmacological agents to decrease LVFP and measured corresponding changes in E/e′ (Table 2).^30^, ^36^, ^42^, ^45^ Despite differences in patient cohorts, agents, and measured indices, all studies concluded that E/e′ does not reflect changes in LVFP.^30^, ^36^, ^42^, ^45^

In 2 studies, the investigators performed serial measurements using implanted hemodynamic measurement devices (Table 2).^41^, ^50^ In 1 study of 15 patients with chronic heart failure with reduced ejection fraction, the investigators found high diagnostic values of E/e′_mean_, E/e′_lateral_, or E/e′_septal_ to predict the elevated mean left atrial pressure (≥15 mm Hg). In their study, E/e′_lateral_ and E/e′_mean_ had no correlation and E/e′_septal_ had only modest correlation (*r*=0.46) with mean left atrial pressure on serial measurements.^41^ In another study of 5 patients with reduced LVEF, the investigators found no correlation between E/e′ and LVFP and no significant diagnostic value of E/e′ to detect elevated LVFP on serial measurements.^50^

### Studies in Specific Cardiac Conditions

Table 3 summarizes study details and results for the 10 studies that included participants with specific cardiac conditions (see Table S5 for more details). In 3 studies, cardiac transplant patients were evaluated.^37^, ^38^, ^39^ In 1 study of 14 transplant patients, serial measurements revealed an excellent correlation between changes in E/e′_mean_ and changes in PCWP.^39^ In contrast, in another study with a larger cohort (N=57), there was no difference in the E/e′ values postexercise despite changes in PCWP.^37^ In another study with similarly large sample cohort, the investigators found low predictive power of exercise E/e′ for identifying elevated PCWP and a modest correlation for the E/e′‐PCWP and ΔE/e′‐ΔPCWP.^38^

**Table 3:** Summary of Studies With Specific Cardiac Conditions

Study	Study Design	Population	N	Intervention	Echo./Cath. Timing	LVFP Change Postintervention	E/e′ Change Postintervention	E/e′‐LVFP Relation Postintervention	ΔE/e′‐ΔLVFP Relation	Prediction of Elevated LVFP Postintervention	Study Summary for Relationship Between E/e′ and LVFP	Comments
Interventions to increase LVFP												
Gurudevan, 2007^47^	Retrospective^a^ Consecutive	Chronic thromboembolic pulmonary hypertension with E<A (NYHA class III–IV), LVEF 66±9%	61	Pulmonary thromboendarterectomy	≤48 hours before and ≤10±6 days after surgery	↑ PCWP	↑ Lateral, Septal	···	···	···	···	On group average, E/e′ does increase reflecting the postsurgery elevation of PCWP
Dalsgaard, 2009^49^	Prospective^a^	Severe aortic stenosis, LVEF 57±8%	28	Supine bicycle	Simultaneous	↑ PCWP	↔ Lateral, Septal	Sign.	n.s.	···	E/e′ does not detect exercise‐induced changes in PCWP in patients with severe aortic stenosis	
Meluzin, 2013^38^	Prospective^a^	Heart transplants, LVEF 65±1%	61	Supine bicycle	Simultaneous	? PCWP	? Mean	Sign.	Sign.	Only for patients with PCWP <15 mm Hg at rest (N=50): AUC 0.74^b^ to detect PCWP ≥25 mm Hg	E/e′ does not sufficiently precise predict the exercise‐induced elevation of PCWP	
Andersen, 2013^48^	Prospective	Post myocardial infarction with LAVI >34 mL, 8<E/e′<15, LVEF 56±7%	61	Supine bicycle	Simultaneous	↑ PCWP	↓ Lateral, Septal, Mean	n.s.	···	···	E/e′ does not reflect exercise‐induced changes in PCWP post‐MI patients with resting E/e′ in the intermediate range	PCWP ↑ and E/e′ ↓
Clemmensen, 2016^37^	Prospective^a^	Heart transplants, LVEF 65±1%	57	Semi‐supine bicycle	Simultaneous	↑ PCWP	↔? Mean	···	···	···	···	E/e′ change did not differ in patients with exercise elevated and not elevated LVFP
Interventions to decrease LVFP												
Hadano, 2007^34^	Prospective^a^ Consecutive	Patients undergoing cardiac surgery, LVEF 40±17%	52	Cardiac surgery	Consequentive	↓ PCWP	↑ Lateral, Septal	Sign.	···	···	E/e′ does correlate with PCWP after cardiac surgery	PCWP ↓ and E/e′ ↑
Serial or repeated measurements												
Sundereswaran, 1998^39^	Prospective	Heart transplants, LVEF 56±12%	14	Repeated measurements at unknown interval	Simultaneous	↓↑ PCWP	↓↑ Mean	···	Sign.	To detect a change in PCWP ≥5 mm Hg: Se./Sp.: 77%/75% if change in E/e′ >2.5	E/e′ does estimate LVFP and track changes in LVFP	
Nagueh, 1999^35^	Prospective^a^	HCM enrolled for ethanol septal reduction	17	Measurements repeated at the end of surgery	Simultaneous	↓↑ Pre‐A	↓↑ Lateral	···	Sign.	···	E/e′ does track changes in LVFP	
Dokainish, 2004^43^	Prospective^a^ Consecutive	ICU or CCU, LVEF 47±18%	9	Measurements repeated at 48 hours	Simultaneous	↓↑ PCWP	↓↑ Mean	···	Sign.		···	E/e′ does track changes in LVFP
Mullens, 2009^51^	Prospective Consecutive	ICU (LVEF<30%)	51	Measurements repeated at 48 hours	Simultaneous	↓↑ PCWP	? Mean	···	n.s.	···	In advanced HF, E/e′ does not reliably predict LVFP	

? indicates not clear from text; ↑ or ↓, statistically significant increase or decrease was measured in the cohort; ↔, no statistically significant change was measured in the cohort; AUC, area under the receiver operating characteristic curve; HCM, hypertrophic cardiomyopathy; ICU/CCU, intensive/critical care unit; Lateral, Septal, and Mean, E/e′_lateral_, E/e′_septal_, and E/e′_mean_; LAVI, left atrial volume index; LVEF, left ventricular ejection fraction; LVFP, left ventricular filling pressure; N, number of patients; NYHA, New York Heart Association; PCWP, pulmonary capillary wedge pressure; pre‐A, left ventricular pre‐A wave pressure; Se./Sp., sensitivity and specificity; Sign./n.s., study reports that correlation coefficient is/is not statistically significant.

a Based on our read.

b Statistically significant value of AUC.

In patients with severe aortic stenosis (N=28)^49^ and in patients with recent myocardial infarction (N=61),^48^ the investigators concluded that E/e′ does not reflect exercise‐induced changes in PCWP.^48^, ^49^ Three studies measured E/e′ and PCWP before and after cardiovascular surgery.^34^, ^35^, ^47^ In 1 study, a strong correlation between E/e′_lateral_ and PCWP was noted before and 30 days after cardiac surgery (coronary artery bypass grafting or aortic valve replacement) (N=52, LVEF 40±17%).^34^ Interestingly in these patients, E/e′_septal_ increased whereas PCWP decreased after surgery.^34^ In a study of hypertrophic cardiomyopathy (N=17), ethanol‐induced septal infarction caused changes in PCWP of either direction, which strongly correlated with changes in E/e′_lateral_.^35^ In patients with chronic thromboembolic pulmonary hypertension (with E<A, NYHA class III‐IV, and preserved LVEF, N=61), both PCWP and E/e′ increased following pulmonary thromboendarterectomy.^47^ Another study reported a strong correlation between individual changes in PCWP and E/e′_mean_ in 9 patients with differing LVEF in the intensive care unit following 2 days of treatment with diuretics and/or inotropes.^43^ However, in 51 patients with decompensated heart because of advanced systolic HF, no correlation was found between changes in E/e′_mean_ and PCWP.^51^

## Discussion

The major findings of our study are that there is lack of robust clinical evidence to support the use of E/e′ in response to physiological and/or pharmacological intervention to estimate LVFP changes and LV diastolic dysfunction. Furthermore, most of the studies are single center with limited sample size with nonuniform study methodologies and data reporting that does not allow for quantitative meta‐analysis of the studies.

Invasive LVFP measurements (primarily LV end diastolic pressure or PCWP as its surrogate) in response to altered physiological conditions provide incremental information about the LV function and stiffness.^4^ In proper context, it can be extremely useful in diagnosing diastolic dysfunction.^4^ Since some studies^5^, ^35^, ^43^, ^52^, ^53^ have suggested that echocardiographic E/e′ can be used to estimate LVFP quantitatively/semiquantitatively, there has been tremendous interest in evaluating changes in E/e′ to physiological and/or pharmacological interventions as a surrogate to changes in LVFP and therefore its potential use in assessing LV diastolic function.^5^, ^6^, ^11^, ^12^, ^13^, ^14^, ^15^, ^16^, ^17^, ^18^, ^19^ Recent meta‐analysis has demonstrated that E/e′ measurements at rest have limited diagnostic accuracy in evaluating LVFP in patients with preserved LVEF.^20^ In the present systematic review, we again noted absence of meaningful correlation (where reported) between E/e′ and LVFP at rest in preserved LVEF (Table S3). In contrast, for the reduced or mixed LVEF group, a stronger correlation between E/e′ and LVFP is reported (Table S4), which may be related to a wider range of E/e′ and LVFP (for instance, see Figure 4 in Nagueh et al^54^). However, other factors may also be playing an important role as Manouras et al^30^ demonstrated a higher correlation for the reduced LVEF group compared to preserved LVEF despite similar LVFP and E/e′ range of values in the 2 cohorts (see Table 3 and Figure 4, Manouras et al^30^). It is interesting to note that the recent American Society of Echocardiography guidelines propose a consensus‐based approach consisting of multiple parameters for evaluating diastolic function in preserved LVEF.^10^ Regarding posthemodynamic changes induced by exercise or physiological interventions, we note that there is no significant correlation between E/e′ and LVFP in preserved LVEF cohorts (Table S3). Moreover, most studies demonstrated worse correlation in mixed or reduced LVEF cohorts after exercise or physiological interventions (Table S4). A similar trend was also noticed in patients with specific cardiac conditions (Table S5).

For evaluating the relationship of change in E/e′ to changes in LVFP in response to exercise or physiological/pharmacological intervention, we find that there are only 2 studies with limited sample size that directly^31^ or indirectly^32^ support the use of E/e′ for the assessment of LVFP changes in HFpEF patients. In 12 patients, Talreja et al^31^ found a promising diagnostic value of specific exercise E/e′ cutoff (>15) to predict elevation of exercise PCWP (>20 mm Hg). Efstratiadis et al^32^ reported concordant reduction of E/e′ and LVFP following nesiritide infusion in 10 patients. Seven other studies^24^, ^26^, ^27^, ^28^, ^29^, ^30^, ^33^ (cumulative N=429) found that E/e′ does not reliably reflect changes in LVFP in response to physiological or pharmacological intervention in preserved EF. For studies that evaluated mixed LVEF groups, results similar to those of the preserved LVEF group were noted. Only 1 study^25^ demonstrated a clinically meaningful relationship and diagnostic characteristics of E/e′ in estimating elevated LVFP with exercise and reduced exercise capacity. No consistent trends were found in other studies with mixed groups. Also, in specific cardiac conditions we did not find consistent trends across the studies. In the present study we did not evaluate the prognostic value or the pathognomonic mechanisms that may be attributed to the lack of reported relationships with exercise or other interventions of changes in E/e′ and LVFP. It is well recognized^7^ that LVFP may increase in diastolic dysfunction on invasive measurements. However, E/e′ measurements did not demonstrate a predictable relationship, which may be attributable to the small sample size of individual studies with relatively heterogeneous LV mechanics. This requires further exploration in future studies.

A number of guidelines/tools such as STARD^55^ and QUADAS^56^ have been developed for evaluating diagnostic test accuracy studies. As evident from our data tables, because of a limited number of studies, limited sample size, and nonuniform methodologies and data reporting, performing such an analysis would not substantially alter our results. Here we are unable to quantify effects of publication bias due to lack of consistent findings and limited studies. However, this is unlikely to affect the overall conclusions.

In summary, our review indicates that there is inadequate evidence for using E/e′ for estimating LVFP changes in response to exercise/other physiological interventions. Well‐designed prospective multicenter studies are required for evaluation and validation before recommending it for clinical and research purposes.

## Sources of Funding

The study was supported by a National Institutes of Health National Heart, Lung, and Blood Institute grant R01‐HL104018. The funding organizations did not have any role in the design and conduct of the study; collection, management, analysis, and interpretation of the data; preparation, review, or approval of the manuscript; and decision to submit the manuscript for publication.

## Disclosures

None.

## Supporting information


**Table S1.** Data Sources and Search Strategy
**Table S2.** Full‐Text Studies Excluded After Evaluation (No Data of Interest)
**Table S3.** Detailed Summary of Studies With Subjects With LVEF ≥50%
**Table S4.** Detailed Summary of Studies With Subjects With Mixed or Reduced LVEF
**Table S5.** Detailed Summary of Studies With Subjects With Specific Cardiac Conditions
