# Supplementary material for: What Is the Evidence That the Tissue Doppler Index E/e′ Reflects Left Ventricular Filling Pressure Changes After Exercise or Pharmacological Intervention for Evaluating Diastolic Function? A Systematic Review
Source: J Am Heart Assoc. 2017 Mar 15;6(3):e004766. doi: 10.1161/JAHA.116.004766 (PMC5524012; doi:10.1161/JAHA.116.004766)
Supplement: Supplementary file 1 — Table S1. Data Sources and Search Strategy Table S2. Full‐Text Studies Excluded After Evaluation (No Data of Interest) Table S3. Detailed Summary of Studies With Subjects With LVEF ≥50% Table S4. Detailed Summary of Studies With Subjects With Mixed or Reduced LVEF Table S5. Detailed Summary of Studies With Subjects With Specific Cardiac Conditions [file JAH3-6-e004766-s001.pdf]

SUPPLEMENTAL MATERIAL

Table S1. Data sources and search strategy.

1. Original search for Sharifov et al. 2016 [1]

PubMed (total of 18791 original citations)	
Search 1	diastol* AND (echo* OR Doppl* OR ultrasound* OR acous*). Limits: English, Journal Article, Humans. Time range: 1/1/1980 - 11/14/2013 (identified **12733** document citations).
Search 2	diastol* AND catheter* AND Doppler* AND pressure. Limits: English, Journal Article, Humans. Time range: 1/1/1970 - 04/28/2014 (identified 738 document citations [**551** new and 187 duplicates])
Search 3	echocardiography AND tissue doppler AND catheterization. Limits: English. Time range: not specified - 02/06/2015 (identified 503 document citations [**291** new and 212 duplicates])
Search 4	((ventric* pressure*) OR "ventricular pressure"[MeSH Terms] OR "ventricular dysfunction"[MeSH Terms]) AND (Doppler* OR E/e* OR "echocardiography, doppler"[MeSH Terms]). Limits: English. Time range: not specified - 02/16/2015 (identified 9776 document citations [**5216** new and 4560 duplicates]). *All studies of our interest, which were selected from the results of Searches 1, 2, and 3 in PubMed, were also identified in the document* citations *of the Search 4*.
Scopus (total of 1580 original citations)	
Search 1	(TITLE-ABS-KEY (echocardiography) OR TITLE-ABS-KEY (tissue Doppler) AND TITLE-ABS-KEY (catheterization) AND DOCTYPE ("ar") AND SUBJAREA (mult OR agri OR bioc OR immu OR phar OR mult OR medi OR nurs OR vete OR dent OR heal) AND (LIMIT-TO (LANGUAGE , "English"). Time range: not specified - 02/06/2015 (identified 512 document citations [**167** new and 345 PubMed duplicates])
Search 2	(TITLE-ABS-KEY (ventric* pressure*) OR TITLE-ABS-KEY (ventricular dysfunction) AND TITLE-ABS-KEY (doppler*) OR TITLE-ABS-KEY (e/e*) OR TITLE-ABS-KEY (echocardiography,doppler)) AND SUBJAREA (mult OR medi OR nurs OR vete OR dent OR heal) AND NOT INDEX (medline) , AND (LIMIT-TO (LANGUAGE , "English")) AND (LIMIT-TO (SUBJAREA , "MEDI")) AND (LIMIT-TO (DOCTYPE , "ar") OR LIMIT-TO (DOCTYPE , "cp")). Time range: not specified - 02/16/2015 (identified **1413** document citations, as not indexed in Medline)
Embase (total of 594 original citations)	
Search 1	(ventric* near/2 pressure or ventric* near/3 'diastolic pressure' or ventric* near/3 'filling pressure' or 'ventricular pressure'/exp or 'ventricular pressure' or 'ventricular dysfunction'/exp or 'ventricular dysfunction' or 'diastolic heart failure'/exp or 'diastolic heart failure' or 'heart failure with normal' or 'heart failure with preserved' and ('doppler' or 'e/e' or 'echocardiography doppler'/exp or 'echocardiography doppler') and ([article]/lim or [article in press]/lim or [conference paper]/lim or [letter]/lim) and [english]/lim and [embase]/lim) and [embase]/lim not [medline]/lim. Time range: not specified - 03/05/2015 (identified **594** document citations, as not indexed in Medline)
Cochrane Library (March 2015) (total of 48 original citations (conference abstracts))	
Search 1	"filling pressure" AND "Doppler" (gives 78 citations [**5** new and 73 duplicates])
Search 2	"filling pressure" AND "E/e" (gives 42 citations [**6** new and 36 duplicates])
Search 3	"diastolic dysfunction" AND "e/e" (gives 46 citations [**10** new and 36 duplicates])
Search 4	"diastolic dysfunction" AND "tissue Doppler" (gives 70 citations [**26** new and 44 duplicates])
Search 5	"diagnostic accuracy" AND "diastolic dysfunction" (gives 3 citations [**0** new and 3 duplicates])
Search 6	"diagnostic accuracy" AND " diastolic heart failure" (gives 0 citations [**0** new and 0 duplicates])
Search 7	"diagnostic accuracy" AND "tissue doppler" (gives 6 citations [**1** new and 5 duplicates])
Search 8	"diagnostic accuracy" AND "E/e" (gives 2 citations [**0** new and 2 duplicates])

2. New search

PubMed (total of 728 citations)	
Search 1	Exercise test AND E/e. Limits: English. Time range: - 09/14/2016 (identified **154** document citations).
Search 2	Exercise AND E/e AND invasive. Limits: English, Time range: - 09/14/2016 (identified **13** document citations)
Search 3	("exercise test"[MeSH Terms] OR ("exercise"[All Fields] AND "test"[All Fields]) OR "exercise test"[All Fields] OR ("exercise"[All Fields] AND "stress"[All Fields] AND "test"[All Fields]) OR "exercise stress

	test"[All Fields]) AND ("Echo"[Journal] OR "echo"[All Fields]) AND invasive[All Fields]. Limits: English. Time range: not specified - 09/15/2016 (identified **83** document citations)
Search 4	((("dobutamine"[MeSH Terms] OR "dobutamine"[All Fields]) AND ("Echo"[Journal] OR "echo"[All Fields]) AND invasive[All Fields]) AND (diastol[All Fields] OR diastola[All Fields] OR diastolc[All Fields] OR diastole[All Fields] OR diastole'[All Fields] OR diastoles[All Fields] OR diastoli[All Fields] OR diastolic[All Fields] OR diastolic'[All Fields] OR diastolic"[All Fields] OR diastoly[All Fields] OR diastolyc[All Fields])) AND ("pressure"[MeSH Terms] OR "pressure"[All Fields])]. Limits: English. Time range: not specified - 09/15/2016 (identified **4** document citations)
Search 5	"cardiac catheterization"[MeSH Terms] AND ((("tissues"[MeSH Terms] OR "tissues"[All Fields] OR "tissue"[All Fields]) AND doppler[All Fields]) OR (early[All Fields] AND mitral[All Fields] AND velocity[All Fields] AND early[All Fields] AND ("diastole"[MeSH Terms] OR "diastole"[All Fields] OR "diastolic"[All Fields]) AND velocity[All Fields] AND mitral[All Fields] AND annulus[All Fields]) OR (early[All Fields] AND mitral[All Fields] AND inflow[All Fields] AND velocity[All Fields] AND mitral[All Fields] AND annular[All Fields] AND early[All Fields] AND ("diastole"[MeSH Terms] OR "diastole"[All Fields] OR "diastolic"[All Fields]) AND velocity[All Fields])) AND ("pressure"[MeSH Terms] OR "pressure"[All Fields]) AND ("diastole"[MeSH Terms] OR ("diastole"[MeSH Terms] OR "diastole"[All Fields] OR "diastolic"[All Fields])) 09/15/2016 (identified **102** document citations)
Search 6	E/e[All Fields] AND (left[All Fields] AND ("heart ventricles"[MeSH Terms] OR ("heart"[All Fields] AND "ventricles"[All Fields]) OR "heart ventricles"[All Fields] OR "ventricular"[All Fields]) AND filling[All Fields] AND ("pressure"[MeSH Terms] OR "pressure"[All Fields])) 09/15/2016 (identified **372** document citations)
Scopus (total of 1391 citations)	
Search 1	(TITLE-ABS-KEY(E/e) AND TITLE-ABS-KEY(left ventricular filling pressure)) AND (LIMIT-TO(DOCTYPE,"ar")) AND (LIMIT-TO(LANGUAGE,"English")) AND (LIMIT-TO(SUBJAREA,"MEDI")) AND (LIMIT-TO(EXACTKEYWORD,"Human")). Time range: not specified - 09/15/2016 (identified **1391** document citations)
Cochrane Library (total of 190 citations)	
Search 1	" left ventricular filling pressure, doppler" (gives **190** trails citations) 09/15/2016

Table S2. Full-text studies excluded after evaluation (no data of interest).

Studies are identified with PMID (if available)

1. 8078825
2. 9052288
3. 12356384
4. 23190400
5. 1905874
6. 1985353
7. 1987211
8. 2214134
9. 2278168
10. 2360494
11. 2498005
12. 2683699
13. 2782257
14. 2871286
15. 2958532
16. 3177175
17. 3209254
18. 7730680
19. 7771173
20. 7817903
21. 8319326
22. 8496538
23. 8606285
24. 8933237
25. 9046493
26. 9237029
27. 9247521
28. 10149211
29. 10969625
30. 11368862
31. 11593199
32. 11884251
33. 12487633
34. 16195393
35. 18325734
36. 24319341
37. 1827808
38. 7780619
39. 8736006
40. 1607511
41. 3392336
42. 8245357
43. 8557907
44. 9015003
45. 10913476
46. 10913478
47. 11279327
48. 11391284
49. 11560356
50. 11770447
51. 11926970
52. 14563593
53. 14652601
54. 15653227
55. 15891754
56. 16128376
57. 16500488
58. 16516591
59. 20197576
60. 19168324
61. 19560662
62. 18612440
63. 18635276
64. 18771556
65. 16716013
66. 17069599
67. 17196474
68. 17451867
69. 17541761
70. 17658724
71. 17884382
72. 18514937
73. 18538465
74. 17560894
75. 21245360
76. 22567531
77. Moladoust H. et al, Echocardiography: A Jrnl. of CV Ultrasound & Allied Tech. (2009) 26 (4), 403-411
78. Said K. et al, The Egyptian Heart Journal (2012) 64, 69-74
79. 11944011
80. 19602775
81. 2816770
82. 7193403
83. 7960266
84. 8890820
85. 12221410
86. 17079190
87. 17484987
88. 18406665
89. 18440343
90. 20117802
91. 22494067
92. 24174962
93. 1760176
94. 1800031
95. 1918702
96. 2629864
97. 2816706
98. 3153793
99. 3532754
100. 3903704
101. 6777405
102. 7561012
103. 7640020
104. 7673761
105. 7802299
106. 8001095
107. 8037096
108. 8184840
109. 8252682
110. 8261053
111. 8771303
112. 8891860
113. 8904686
114. 9043850
115. 9104907
116. 9137220
117. 9203493
118. 9424066
119. 9950969
120. 10230946
121. 10441218
122. 10980082
123. 11158951
124. 11407738
125. 11408426
126. 11433812
127. 11433813
128. 11433824
129. 11482709
130. 11490324
131. 11550110
132. 11585994
133. 11593203
134. 11696830
135. 11796872
136. 11809440
137. 11917193
138. 12094170
139. 12707119
140. 12714167
141. 12766750
142. 12804750
143. 12848693
144. 12940700
145. 14640103
146. 14641374
147. 14652600
148. 14670073

149. 14672750
150. 14717717
151. 14752488
152. 15172419
153. 15309696
154. 15476639
155. 15488086
156. 15948097
157. 16014646
158. 16223980
159. 16284230
160. 16344121
161. 16434758
162. 16575023
163. 16949491
164. 17207727
165. 17313636
166. 17390199
167. 17488411
168. 18198205
169. 18471459
170. 18597919
171. 19203992
172. 20058507
173. 20553318
174. 20625213
175. 20682947
176. 20970305
177. 21262980
178. 21316304
179. 21683506
180. 22577437
181. 22739787
182. 23146480
183. 23194487
184. 23689521
185. 23879336
186. 22066607
187. 2705380
188. 9183590
189. 11502702
190. 12167386
191. 15979445
192. 16174119
193. 16803936
194. 16970713
195. 24839086
196. 10440167
197. 11175032
198. 11595603
199. 12714167
200. 15307890
201. 16174119
202. 24621836
203. 24839086
204. 24943993
205. 24958524
206. 25249511
207. 25441329
208. 25510308
209. 21602549
210. 23883877
211. 24869961
212. 24902871
213. Cong T. et al, Experimental & Clinical Cardiology (2014) 20 (1), 2479-2490
214. 2296893
215. 2672760
216. 15325936
217. 9247519
218. 3177234
219. 2360518
220. 2913110
221. 7710749
222. 9385913
223. 11153819
224. 15389248
225. 18091642
226. 20609653
227. 21723693
228. 11179524
229. 15084546
230. 17291934
231. 22645191
232. 23316319
233. 11796546
234. 11263606
235. 11263607
236. 11270316
237. 11585994
238. 23582091
239. 10636281
240. 10849514
241. 10910486
242. 3280641
243. 1869739
244. 11121596
245. 21718357
246. 18636341
247. 22473456
248. 23555178
249. 11093099
250. 22632828
251. 25611697
252. 23074579
253. 24626519
254. 24995376
255. 25414078
256. Wang W. et al, Acta Cardiol Sin (2012), 28, 206-215
257. Ahn J. et al, e-Herz (2013), DOI 10.1007/s00059-013-4010-0
258. 21718351
259. 27354047
260. 27573595
261. Ma H. et al. Ultrasound in Med. & Biol (2016) http://dx.doi.org/10.1016/j.ultrasmedbio.2015.09.022
262. 25533754
263. 27582773
264. 26343250
265. 12578874
266. 15637491
267. 18025528
268. 18222643
269. 18653572
270. 20139439
271. 20394874
272. 21478380
273. 22939039
274. 23549512
275. 23824244
276. 25532095
277. 25747153
278. 25956143
279. 26603966
280. 20543134
281. 25044440
282. 25393338
283. 20406766
284. 20705267
285. 21685198
286. 25342738
287. 25450014
288. 26082167
289. 26216649
290. 25161181
291. 26914917
292. 20418368
293. 25701392
294. 27021934
295. 21865226
296. 22661507
297. 23002243
298. 25468660
299. 18413502
300. 23103948
301. 22705767

Table S3. Detailed Summary of studies with subjects with LVEF ≥50%.

Study	N	Subjects	Intervention	Echo./ Cath. timing	LVFP values at baseline and post intervention (mmHg)	E/e' values at baseline and post intervention	E/e'-LVFP relation (r)		ΔE/e' - ΔLVFP relation	Prediction of elevated LVFP			Comments
							Baseline	Intervention		Baseline	Intervention	Δ E/e'	
**Interventions to increase LVFP**													
Firstenberg, 2000[2]	7	Healthy volunteers, LVEF 72±4%, 37±9 yr.	Saline infusion	Simult.	PCWP:<br>10.7±1.9 – 20.0±3.3*	Lateral:<br>4.6±0.3 - 6.1±1.3<br>Septal:<br>6.5±0.8 – 7.6±1.3	NA	NA	NA	NA	NA	NA	E/e' **does not** change despite significant elevation of LVFP
Talreja, 2007[3]	12	Patients with exertional dyspnea (NYHA class II-III)	Supine bicycle	Simult.	PCWP:<br>14±4 - 22±10*	Septal:<br>11.7±0.5 – 14.5±0.6	NA	NA	NA	NA	*†Se./Sp:*<br>83%/100% to predict PCWP≥20 mm Hg if E/e'>15	NA	
Maeder, 2010[4]	8-22	*HFpEF (N=14)*<br>LVEF 63±6%, 69±10 yr.<br>*Controls (N=8)*<br>LVEF 62±5%, 61±12 yr.<br>*Total cohort (N=*22)	supine bicycle	Simult.	PCWP:<br>10±4 -23±6*<br><br>10±4 -20±7*<br><br>NA	Septal:<br>13±4.1–9.5±3.4*<br><br>9.5±3.4 -†9.4±3.4<br><br>NA	 NA<br><br>NA<br><br>0.19	 NA<br><br>NA<br><br>0.22	 NA	 n.s.	 NA	 NA	
Choi, 2016[5]	181	HFpEF (LVEF>50%, at rest 8<E/e´<15, E/A<1, or e´<8 cm/s)	Passive and active leg-raise	Simult.	LVEDP<br>18.0±5.5 – 24.9±8.3*<br>Pre-A<br>9.0±3.1 – 11.2±8.7*	Septal:<br>10.0±2.5 - no change	NA	NA	NA	NA	NA	NA	E/e' remained unchanged despite increase of LVFP
**Interventions to decrease LVFP**													
Chan, 2011[6]	16	Patients w/out significant CAD, LVEF>50%, 57±8 yr.	Dobutamine i.v.	Simult.	LVEDP:<br>19.9±4.1 – 8.1±6.2*<br>LVMDP:<br>12.3±2.6 – 9.0±2.3*	Lateral:<br>7.5±1.9 – 7.9±3.5<br>Septal:<br>8.7±2.2 – 8.3±3.1	NA	Lateral:<br>n.s.<br>Septal:<br>n.s.	Lateral:<br>n.s.<br>Septal:<br>n.s.	NA	NA	NA	
Manouras, 2013[7]	38	Patients with stable angina and/or exertional dyspnea, LVEF>55% (60±4.5%)	Nitroglycerin i.v.	Simult.	LVEDP:<br>20.2±6.5 - 15.5±5.7*<br>Pre-A:<br>14.2±4.7 - 10.7±4.3*	Lateral:<br>NA<br><br>Septal:<br>NA<br><br>Mean:<br>8.9±2.9 – 8.2±2.5	Lateral:<br>0.33*<br>0.4*<br>Septal:<br>0.03<br>0.02<br>Mean:<br>0.18<br>0.21	Lateral:<br>0.14<br>0.15<br>Septal:<br>0.11<br>0.16<br>Mean:<br>0.13<br>0.08	Lateral:<br>NA<br><br>Septal:<br>NA<br><br>Mean:<br>n.s.<br>0.08	NA	NA	NA	After NTG, number of patients with LVEF>55% changed from 38 to 52<br>B-only for patients with LVFP decreased to normal (pre-A ≤12 mm HG) after NTG
Firstenberg, 2000[2]	7	Healthy volunteers, LVEF 72±4%, 37±9 yr.	lower-body negative pressure	Simult.	PCWP<br>10.3±2.0 – 4.0±1.4*	Lateral:<br>6.2±1.5 – 5.5±1.6<br>Septal:<br>7.1±2.2 – 7.4±2.0	NA	NA	NA	NA	NA	NA	E/e' **does not** change despite decrease of LVFP
Efstratiadis, 2009[8]	10	HFpEF patients, LVEF 64±7%, 61±10 yr.	Nesiritide i.v.	Conseq.	PCWP:<br>19±9 - 11±7*<br>LVEDP:<br>18.7±6.1 – 10.4±4.9*	Lateral:<br>10.8±3.7 – 7.9±3.0*	NA	NA	NA	NA	NA	NA	E/e' and LVFP decreased. However, ΔE/e' **does not**

													correlate with ΔLVFP (see Weeks, 2008[9])
Santos, 2015[10]	118	Patients with unexplained dyspnea, LVEF 63±8%, 57 (40-79) yr.	From supine to upright position	Conseq. for supine, Simult. for upright	PCWP: 12±5 – 7±4*	Lateral: 7.3±3.4 – 8.5±3.2<br><br>Septal: 9.7±3.6 – 10.3±3.7<br><br>Mean: 8.2±3.4 – 9.2±3.3	Lateral: 0.30* (N=89)<br><br>Septal: 0.41* (N=91)<br><br>Mean: 0.36* (N=88)	Lateral: 0.03 (N=61)<br><br>Septal: 0.19 (N=80)<br><br>Mean: 0.10 (N=59)	Lateral: -0.07 (N=52)<br><br>Septal: 0.07 (N=64)<br><br>Mean: 0.04 (N=50)	Lateral: *AUC* 0.62 (95%CI, 0.46-0.78, N=89)<br><br>Septal: *AUC* 0.67 (95%CI, 0.53-0.81, N=93)<br><br>Mean: *AUC* 0.65 (95%CI, 0.50-0.79, N=88)	NA	NA	E/e' does not accurately estimate PCWP, does not identify patients with elevated PCWP. Positional change in E/e' does not reflect change in PCWP.
**Analysis of combined measurements from baseline and during intervention**													
Firstenberg, 2000[2]	7	Healthy volunteers, LVEF 72±4%, 37±9 yr.	lower-body negative pressure –saline infusion	Simult.	PCWP: 4.0±1.4 – 20.0±3.3*	Lateral: 5.5±1.6 – 6.1±1.3<br>Septal: 7.4±2.0– 7.6±1.3	Lateral: 0.17<br>Septal: 0.14<br>(30 measurements)		NA	NA	NA	NA	E/e' **does not** change despite significant LVFP changes. E/e' did not correlate with LVFP.
Bhella, 2011[11]	47	Outpatients HFpEF (N=11, 73±7 yr.) and healthy old (N=24, 69±3 yr.) and young (N=12, 32±9 yr.) controls, LVEF >50%	lower-body negative pressure –saline infusion	Simult.	PCWP: Range: 0.8 - 28.8	Mean: Range:~2.5 - 20	NA		NA	NA	NA	NA	R^2 for individual linear regression ranged from 0.00 to -0.94. Slopes ranged from -6.76 to 11.03.

N – number of patients with LVEF>50% (not always a total N of patients in the study); Values are mean±SD. Lateral, Septal, and Mean – $E/e'_{lateral}$, E/e'_{septal}, and E/e'_{mean}; LVEF=left ventricular ejection fraction; LVFP=left ventricular filling pressure; LVEDP=left ventricular end diastolic pressure; pre-A=left ventricular pre- A wave pressure; LVMDP=left ventricular mean diastolic pressure; LAP=left atrial pressure; PCWP=pulmonary capillary wedge pressure; CAD=coronary artery disease; HFpEF=heart failure with preserved ejection fraction; AUC= area under the receiver operating characteristic curve; Se./Sp. – Sensitivity and Specificity; n.s. – study reports that correlation coefficient is not statistically significant; NA – not available (not reported). * statistically significant; † our assessment made from the study data.

Table S4. Detailed summary of studies with subjects with mixed or reduced LVEF.

Study	N	Subjects	Intervention	Echo./ Cath. timing	LVFP values at baseline and post intervention (mmHg)	E/e' values at baseline and post intervention	E/e'-LVFP relation (r)		ΔE/e' - ΔLVFP relation	Prediction of elevated LVFP			Comments
							Baseline	Intervention		Baseline	Intervention	Δ E/e'	
**Interventions to increase LVFP**													
Burgess, 2006[12]	37	Unselected patients, LVEF NA (9 patients with LVEF<45%), 61±9 yr.	Single leg supine cycle	Simult.	LVMDP:<br>11.2±6.6 – 14.5±7.9<br>(?)	Septal:<br>11.7±4.4 – 13.4±6.2<br>(?)	0.67*	0.59*	NA	NA	AUC: 0.89* To predict LVMDP>15 mmHg *Se./Sp.:* 73%/96% to predict LVMDP>15 mm Hg if E/e'>13	NA	
Gibby, 2013[13]	37	LVEF 56±12%, 61±9 yr			NA	NA	NA	NA	NA	NA	*Se./Sp.:* 67%/95% to predict LVMDP>15 mm Hg if E/e'>13	NA	
Yamada, 2014[14]	22	Patients with various chronic cardiac diseases, LVEF 58±14%	Leg-positive pressure at 90 mm Hg	Simult.	LVEDP:<br>11.6±4.1 – 16.3±5.8*<br>Pre-A:<br>7.1±2.8 – 9.6±4.2*	Lateral:<br>9.2±4.0 – 11.6±7.0*	NA	NA	NA	NA	NA	NA	
Marchandise, 2014[15]	40	LV systolic dysfunction, LVEF 27±11%, 54±12 yr	Semi-supine bicycle	Simult.	PCWP:<br>15±7 - 24±9*	Lateral:<br>14.3±7.4 – 10.8±4.1*<br>Septal:<br>17.7±7.6 – 15.5±6.6*<br>Mean:<br>14.5±5.3 – 12.3±4.1*	Lateral:<br>0.14<br>Septal:<br>0.75*<br>Mean:<br>0.44	Lateral:<br>0.40*<br>Septal:<br>0.57*<br>Mean:<br>0.52*	NA	Lateral:<br>NA<br>Septal:<br>*Se./Sp.:*<br>91%/77% to predict PCWP>12 mm Hg if E/e'>15<br>Mean:<br>NA	NA	NA	
**Interventions to decrease LVFP**													
Weeks, 2008[9]<br><br>Efstratiadis, 2009[8]	25	10 HFpEF and 15 HFrEF, LVEF 45±19%/ 60±11 yr.	Nesiritide i.v.	Conseq.	PCWP:<br>17±8 - 10±6*<br>LVEDP:<br>18.7±7.1 – 9.8±4.9*	Lateral:<br>9.3±4 - 7±3.5*	PCWP: 0.29* (combined before and after nesiritide)<br>LVEDP: 0.35* (combined before and after nesiritide)		PCWP:<br>0.12<br>LVEDP:<br>0.01	NA	NA	NA	
Manouras, 2013[7]	65	Stable angina and/or exertional dyspnea, LVEF >40%, 66±9 yr.	Nitroglycerin i.v.	Simult.	LVEDP:<br>Approx. 21 – 16 (not reported but can be estimated from subgroup datasets, changes in subgroups are significant)	Lateral:<br>NA<br>Septal:<br>NA<br>Mean:<br>n.s.	Lateral:<br>0.47*<br>Septal:<br>0.31*<br>Mean:<br>0.41*	Lateral:<br>0.25*<br>Septal:<br>025*<br>Mean:<br>0.25*	NA	Lateral:<br>NA<br>Septal:<br>NA<br>Mean:<br>NA	Lateral:<br>NA<br>Septal:<br>NA<br>Mean:<br>n.s.	Lateral:<br>NA<br>Septal:<br>NA<br>Mean:<br>n.s.	

					Pre-A:<br>Approx. 15 – 11 (not reported but can be estimated from subgroup datasets, changes in subgroups are significant)	Lateral:<br>NA<br>Septal:<br>NA<br>Mean:<br>n.s.	Lateral:<br>0.54*<br>Septal:<br>0.34*<br>Mean:<br>0.48*	Lateral:<br>0.26*<br>Septal:<br>025*<br>Mean:<br>0.27*		Lateral: AUC<br>0.71±0.08*<br>Septal: AUC<br>0.62±0.09<br>Mean: AUC<br>0.70±0.089<br>to predict<br>Pre-A>12<br>mmHg	to predict<br>LVEDP>16 mmHg<br><br>Lateral: AUC<br>0.59±0.08<br>Septal: AUC<br>0.59±0.08<br>Mean: AUC<br>0.59±0.08<br>to predict Pre-A>12 mmHg	to predict LVEDP >16 mmHg<br><br>Lateral:<br>NA<br>Septal:<br>NA<br>Mean:<br>n.s.<br>to predict Pre-A>12 mmHg	
Egstrup, 2013[16]	14	Chronic HFrEF outpatients, LVEF 36±8% (<45%), 65±8 yr.	Dobutamine i.v.	Simult.	PCWP:<br>16.6±8.3 – 14.2±9.2	Septal:<br>15.6±7.6 – 14.0±5.2	Septal:<br>0.64*	Septal:<br>0.40	NA	NA	NA	NA	
Chiang, 2014[17]	60	Suspected CAD, LVEF 43±16%, 62.6±11.8 yr.	Glyceryl trinitrate i.v.	Simult.	LVEDP:<br>31.3±12.1 – 15.8±7.4*<br>Pre-A:<br>21.9±8.5 – 11.3±5.4*	Septal:<br>13.5±4.6 – 11.1±3.7*	Septal:<br>0.29*	Septal:<br>NA<br>(P=0.51)	NA	NA	NA	NA	
**Serial or repeated measurements**													
Ritzema, 2011[18]	15	Ambulant chronic HFrEF, LVEF 32±12%/ approx. 71 yr.	Serial measurements with implantable pressure sensor	Simult.	LAP:<br>17.3±8 (baseline)	Lateral:<br>16.6±9.3 (baseline)<br><br>Septal:<br>21±10.4 (baseline)<br><br>Mean:<br>NA	Lateral:<br>0.15 (60 measurements)<br><br>Septal:<br>0.46* (60 measurements)<br><br>Mean:<br>0.13 (60 measurements		Lateral:<br>NA<br><br>Septal:<br>0.46*<br><br>Mean:<br>NA	Lateral:<br>*AUC*= 0.90* (60 measurements)<br>*Se./Sp.*: 73%/87% if E/e'≥12<br>Septal:<br>*AUC*= 0.90* (60 measurements)<br>*Se./Sp.*: 84%/91% if E/e'≥15<br>*Mean:*<br>AUC=0.95* (60 measurements)<br>*Se./Sp.*: 84%/96% if E/e'≥14<br>to detect LAP≥15 mm Hg		NA	
Goebel, 2011[19]	5	Patients scheduled for aortocoronary bypass surgery, LVEF <35% (25%-35%)/ NA yr.	Serial measurements with implantable pressure sensor	Simult.	LVEDP<br>Values of individual measurements range from 5 to 25 (median 17)<br><br>LVMDP<br>Values of individual measurements range from 7 to 29 (median 20)	Lateral:<br>NA<br>Septal:<br>NA<br>Mean:<br>NA<br><br>Lateral:<br>NA<br>Septal:<br>NA<br>Mean:<br>NA	Lateral:<br>0.35 (21 measurements)<br>Septal:<br>0.16 (21 measurements<br>Mean:<br>0.35 (21 measurements<br><br>Lateral:<br>0.35 (21 measurements)<br>Septal:<br>0.29 (21 measurements<br>Mean:<br>0.35 (21 measurements		NA	Lateral:<br>AUC=0.57 (21 measurements)<br>Septal:<br>AUC=0.63 (21 measurements<br>Mean:<br>AUC=0.67 (21 measurements<br>To detect LVEDP>15 mmHg<br><br>Lateral:<br>AUC=0.76 (21 measurements)<br>Septal<br>AUC=0.76 (21 measurements)<br>Mean:<br>AUC=0.82* (21 measurements<br>To detect LVMDP>12 mmHg		NA	

N – number of patients; Values are mean±SD. Lateral, Septal, and Mean – $E/e'_{lateral}$, E/e'_{septal}, and E/e'_{mean}; LVEF=left ventricular ejection fraction; LVFP=left ventricular filling pressure; LVEDP=left ventricular end diastolic pressure; pre-A=left ventricular pre- A wave pressure; LVMDP=left ventricular mean diastolic

pressure; LAP=left atrial pressure; PCWP=pulmonary capillary wedge pressure; CAD=coronary artery disease; HFpEF=heart failure with preserved ejection fraction; HFrEF=heart failure with reduced ejection fraction; AUC= area under the receiver operating characteristic curve; Se./Sp. – Sensitivity and Specificity; n.s. – study reports that correlation coefficient is not statistically significant; ? – not clear from text; NA – not available (not reported). * statistically significant.

Table S5. Detailed summary of studies with subjects with specific cardiac conditions.

Study	N	Subjects	Intervention	Echo./ Cath. timing	LVFP values at baseline and post intervention (mmHg)	E/e' values at baseline and post intervention	E/e'-LVFP relation (r)		ΔE/e' - ΔLVFP relation	Prediction of elevated LVFP			Comments
							Baseline	Intervention		Baseline	Intervention	Δ E/e'	
**Interventions to increase LVFP**													
Gurudevan, 2007[20]	61	Chronic thromboembolic pulmonary hypertension with E < A (NYHA class III-IV), LVEF 66±9%, 57±13 yr.	Pulmonary thromboendarterectomy	≤48h before and ≤10±6 days after surgery	PCWP: 9.2±3.2 – 10.6±3.8*	Lateral: 6.2±2.2 – 7.4±3.2* Septal: 7.8±3.8 – 10.9±4.6*	NA	NA	NA	NA	NA	NA	
Dalsgaard, 2009[21]	28	Severe aortic stenosis, LVEF 57±8%,70±8 yr.	Supine bicycle	Simult.	PCWP: 18±8 - 39±10*	Lateral: 14±4 – 15±4 (P=0.05) Septal: 19±6 – 19±6	Lateral: 0.67* Septal: 0.72*	Lateral: 0.47* Septal: 0.66*	Lateral: 0.09 Septal: 0.29	NA	NA	NA	
Meluzin, 2013[22]	61	Heart transplants, LVEF 65±1%	Supine bicycle	Simult.	PCWP: Individual change approx. 15±1.1	Mean: Individual change approx. 2.0±0.3	Mean: 0.48*	Mean: 0.42*	Mean: 0.45*	NA	Only for patients with PCWP <15 mmHg at rest (N=50): *AUC* 0.74* to detect PCWP ≥25 mmHg. At exercise E/e' ≥8.5 Se./Sp.: 64%/84%	NA	
Andersen, 2013[23]	61	Post Myocardial Infarction with LAVI >34 ml, 8<E/e'<15, LVEF 56±7% (>45%), 62±8 yr.	Supine bicycle	Simult.	PCWP: 13±4 - 28±8 (4METs)- 33±8* (Peak)	Lateral: NA Septal: NA Mean: 10.5±1.7 – 9.9±2.7 (4METs) - 9.3±2.3* (Peak)	Lateral: 0.18 Septal: 0.16 Mean: 0.20	Lateral: 0.27*(4METs), 0.17 (Peak) Septal: 0.22 (4METs), 0.11 (Peak) Mean: 0.26 (4METs), 0.16 (Peak)	NA	NA	NA	NA	
Clemmensen, 2016[24]	57	Heart transplants, LVEF 65±1% Group 1: *PCWP <15 mm Hg at rest and <25 mm Hg at peak exercise* (LVEF 63±9%), 52±14 yr., N=31) Group 2: PCWP ≥15 mm Hg at rest or ≥25 mm Hg at peak exercise (LVEF 59±10%, 43±13 yr., N=26)	Semi-supine bicycle	Simult.	PCWP: NA 8±2 - 18±4* 14±5 - 34±4*	Mean: NA 8±3 - 10±3 (P=?) 13±7 - 14±6 (P=?)	NA	NA	NA	NA	NA	NA	

**Interventions to decrease LVFP**													
Hadano, 2007[25]	52	Patients undergoing cardiac surgery, LVEF 53±15%, 66±10 yr.	Cardiac surgery	Conseq.	PCWP: 10.8±5.5 – 7.9±3.4*	Lateral: 10.1±4.3 – 7.5±3.1* Septal: 10.7±4.2 – 12.2±5.6*	Lateral: 0.79* Septal: 0.67*	Lateral: 0.69* Septal: 0.44* 30±15 days after surgery	NA	NA	NA	NA	
**Serial or repeated measurements**													
Sundereswaran, 1998[26]	14	Heart transplants, LVEF 56±12%	Repeated measurements at unknown interval	Simult.	PCWP: Individual change 2±6 (range -8 to 13)	Mean: Individual change NA	Mean: NA	Mean: NA	Mean: 0.87*	NA	NA	To detect a change in PCWP ≥5 mm Hg: Se./Sp.: 77%/75% if ΔE/e' >2.5	
Nagueh, 1999[27]	17	HCM enrolled for ethanol septal reduction, for total cohort of 35 patients: LVEF NA, 52±15 yr.	Measurements repeated at the end of surgery	Simult.	Pre-A: Individual changes from -5 to 14	Lateral: Individual changes from -6.1 to 12	NA	NA	0.8*	NA	NA	NA	
Dokainish, 2004[28]	9	ICU or CCU, LVEF 47±18%	Measurements repeated at 48 h	Simult.	PCWP: Individual changes from -8 to 9	Mean: Individual changes from -9 to 4	NA	NA	0.87*	NA	NA	NA	No ΔPCWP-ΔE/e' correlation was for LVEF>50% (N=3)
Mullens, 2009[29]	51	ICU (LVEF<30%)	Measurements repeated at 48 h	Simult.	PCWP: Individual changes from -24 to 16	Mean: NA	NA	NA	0.23 (P=0.1)	NA	NA	NA	

N – number of patients; Values are mean±SD. Lateral, Septal, and Mean – $E/e'_{lateral}$, E/e'_{septal}, and E/e'_{mean}; LVEF=left ventricular ejection fraction; LVFP=left ventricular filling pressure; pre-A=left ventricular pre- A wave pressure; PCWP=pulmonary capillary wedge pressure; AUC= area under the receiver operating characteristic curve; NYHA=New York Heart Association; LAVI=left atrial volume index; HCM=hypertrophic cardiomyopathy; ICU/CCU=intensive/critical care unit; 4METs=four metabolic equivalents of task; Se./Sp. – Sensitivity and Specificity; n.s. – study reports that correlation coefficient is not statistically significant; ? – not clear from text; NA – not available (not reported). * statistically significant.

Supplemental References:

1. Sharifov OF, Schiros CG, Aban I, Denney TS, Gupta H. Diagnostic Accuracy of Tissue Doppler Index E/e' for Evaluating Left Ventricular Filling Pressure and Diastolic Dysfunction/Heart Failure With Preserved Ejection Fraction: A Systematic Review and Meta-Analysis. *J Am Heart Assoc.* 2016;5:e002530.

2. Firstenberg MS, Levine BD, Garcia MJ, Greenberg NL, Cardon L, Morehead AJ, Zuckerman J, Thomas JD. Relationship of echocardiographic indices to pulmonary capillary wedge pressures in healthy volunteers. *J Am Coll Cardiol.* 2000;36:1664-1669.

3. Talreja DR, Nishimura RA, Oh JK. Estimation of left ventricular filling pressure with exercise by Doppler echocardiography in patients with normal systolic function: a simultaneous echocardiographic-cardiac catheterization study. *J Am Soc Echocardiogr*. 2007;20:477-479.

4. Maeder MT, Thompson BR, Brunner-La Rocca HP, Kaye DM. Hemodynamic basis of exercise limitation in patients with heart failure and normal ejection fraction. *J Am Coll Cardiol.* 2010;56:855-863.

5. Choi S, Shin JH, Park WC, Kim SG, Shin J, Lim YH, Lee Y. Two Distinct Responses of Left Ventricular End-Diastolic Pressure to Leg-Raise Exercise in Euvolemic Patients with Exertional Dyspnea. *Korean Circ J.* 2016;46:350-364.

6. Chan AK, Govindarajan G, Del Rosario ML, Aggarwal K, Dellsperger KC, Chockalingam A. Dobutamine stress echocardiography Doppler estimation of cardiac diastolic function: a simultaneous catheterization correlation study. *Echocardiography*. 2011;28:442-447.

7. Manouras A, Nyktari E, Sahlen A, Winter R, Vardas P, Brodin LA. The value of E/Em ratio in the estimation of left ventricular filling pressures: impact of acute load reduction: a comparative simultaneous echocardiographic and catheterization study. *Int J Cardiol.* 2013;166:589-595.

8. Efstratiadis S, Michaels AD. Acute hemodynamic effects of intravenous nesiritide on left ventricular diastolic function in heart failure patients. *J Card Fail.* 2009;15:673-680.

9. Weeks SG, Shapiro M, Foster E, Michaels AD. Echocardiographic predictors of change in left ventricular diastolic pressure in heart failure patients receiving nesiritide. *Echocardiography.* 2008;25:849-855.

10. Santos M, Rivero J, McCullough SD, West E, Opotowsky AR, Waxman AB, Systrom DM, Shah AM. E/e' Ratio in Patients With Unexplained Dyspnea: Lack of Accuracy in Estimating Left Ventricular Filling Pressure. *Circ Heart Fail.* 2015;8:749-756.

11. Bhella PS, Pacini EL, Prasad A, Hastings JL, Adams-Huet B, Thomas JD, Grayburn PA, Levine BD. Echocardiographic indices do not reliably track changes in left-sided filling pressure in healthy subjects or patients with heart failure with preserved ejection fraction. *Circ Cardiovasc Imaging*. 2011;4:482-489.

12. Burgess MI, Jenkins C, Sharman JE, Marwick TH. Diastolic stress echocardiography: hemodynamic validation and clinical significance of estimation of ventricular filling pressure with exercise. *J Am Coll Cardiol.* 2006;47:1891-1900.

13. Gibby C, Wiktor DM, Burgess M, Kusunose K, Marwick TH. Quantitation of the diastolic stress test: filling pressure vs. diastolic reserve. *Eur Heart J Cardiovasc Imaging*. 2013;14:223-227.

14. Yamada H, Kusunose K, Nishio S, Bando M, Hotchi J, Hayashi S, Ise T, Yagi S, Yamaguchi K, Iwase T, Soeki T, Wakatsuki T, Sata M. Pre-load stress echocardiography for predicting the prognosis in mild heart failure. *JACC Cardiovasc Imaging*. 2014;7:641-649.

15. Marchandise S, Vanoverschelde JL, D'Hondt AM, Gurne O, Vancraeynest D, Gerber B, Pasquet A. Usefulness of tissue Doppler imaging to evaluate pulmonary capillary wedge pressure during exercise in patients with reduced left ventricular ejection fraction. *Am J Cardiol.* 2014;113:2036-2044.

16. Egstrup M, Gustafsson I, Andersen MJ, Kistorp CN, Schou M, Tuxen CD, Moller JE. Haemodynamic response during low-dose dobutamine infusion in patients with chronic systolic heart failure: comparison of echocardiographic and invasive measurements. *Eur Heart J Cardiovasc Imaging*. 2013;14:659-667.

17. Chiang SJ, Daimon M, Ishii K, Kawata T, Miyazaki S, Hirose K, Ichikawa R, Miyauchi K, Yeh MH, Chang NC, Daida H. Assessment of elevation of and rapid change in left ventricular filling pressure using a novel global strain imaging diastolic index. *Circ J.* 2014;78:419-427.

18. Ritzema JL, Richards AM, Crozier IG, Frampton CF, Melton IC, Doughty RN, Stewart JT, Eigler N, Whiting J, Abraham WT, Troughton RW. Serial Doppler echocardiography and tissue Doppler imaging in the detection of elevated directly measured left atrial pressure in ambulant subjects with chronic heart failure. *JACC Cardiovasc Imaging.* 2011;4:927-934.

19. Goebel B, Luthardt E, Schmidt-Winter C, Otto S, Jung C, Lauten A, Figulla HR, Gummert JF, Poerner TC. Echocardiographic evaluation of left ventricular filling pressures validated against an implantable left ventricular pressure monitoring system. *Echocardiography.* 2011;28:619-625.

20. Gurudevan SV, Malouf PJ, Auger WR, Waltman TJ, Madani M, Raisinghani AB, DeMaria AN, Blanchard DG. Abnormal left ventricular diastolic filling in chronic thromboembolic pulmonary hypertension: true diastolic dysfunction or left ventricular underfilling? *J Am Coll Cardiol.* 2007;49:1334-1339.

21. Dalsgaard M, Kjaergaard J, Pecini R, Iversen KK, Kober L, Moller JE, Grande P, Clemmensen P, Hassager C. Left ventricular filling pressure estimation at rest and during exercise in patients with severe aortic valve stenosis: comparison of echocardiographic and invasive measurements. *J Am Soc Echocardiogr*. 2009;22:343-349.

22. Meluzin J, Hude P, Krejci J, Spinarova L, Podrouzkova H, Leinveber P, Dusek L, Soska V, Tomandl J, Nemec P. Noninvasive prediction of the exercise-induced elevation in left ventricular filling pressure in post-heart transplant patients with normal left ventricular ejection fraction. *Exp Clin Cardiol*. 2013;18:63-72.

23. Andersen MJ, Ersboll M, Gustafsson F, Axelsson A, Hassager C, Kober L, Boesgaard S, Pellikka PA, Moller JE. Exercise-induced changes in left ventricular filling pressure after myocardial infarction assessed with simultaneous right heart catheterization and Doppler echocardiography. *Int J Cardiol.* 2013;168:2803-2810.

24. Clemmensen TS, Eiskjaer H, Logstrup BB, Mellemkjaer S, Andersen MJ, Tolbod LP, Harms HJ, Poulsen SH. Clinical features, exercise hemodynamics, and determinants of left ventricular elevated filling pressure in heart-transplanted patients. *Transpl Int.* 2016;29:196-206.

25. Hadano Y, Murata K, Tanaka N, Muro A, Akagawa E, Tanaka T, Kunichika H, Matsuzaki M. Ratio of early transmitral velocity to lateral mitral annular early diastolic velocity has the best correlation with wedge pressure following cardiac surgery. *Circ J.* 2007;71:1274-1278.

26. Sundereswaran L, Nagueh SF, Vardan S, Middleton KJ, Zoghbi WA, Quinones MA, Torre-Amione G. Estimation of left and right ventricular filling pressures after heart transplantation by tissue Doppler imaging. *Am J Cardiol.* 1998;82:352-357.

27. Nagueh SF, Lakkis NM, Middleton KJ, Spencer WH, 3rd, Zoghbi WA, Quinones MA. Doppler estimation of left ventricular filling pressures in patients with hypertrophic cardiomyopathy. *Circulation.* 1999;99:254-261.

28. Dokainish H, Zoghbi WA, Lakkis NM, Al-Bakshy F, Dhir M, Quinones MA, Nagueh SF. Optimal noninvasive assessment of left ventricular filling pressures: a comparison of tissue Doppler echocardiography and B-type natriuretic peptide in patients with pulmonary artery catheters. *Circulation.* 2004;109:2432-2439.

29. Mullens W, Borowski AG, Curtin RJ, Thomas JD, Tang WH. Tissue Doppler imaging in the estimation of intracardiac filling pressure in decompensated patients with advanced systolic heart failure. *Circulation.* 2009;119:62-70.
